# Lightning safety awareness level in Malaysia

**DOI:** 10.12688/f1000research.73064.2

**Published:** 2021-11-04

**Authors:** Khairul Nazri, Siow Chun Lim, Chandima Gomes

**Affiliations:** 1 Faculty of Engineering (FOE), Multimedia University (MMU), Cyberjaya, Selangor, 63100, Malaysia; 2 School of Electrical & Information Engineering, University of the Witwatersrand, Johannesburg, South Africa

**Keywords:** Lightning, lightning safety, public belief, Malaysia, lightning myth

## Abstract

**Introduction**: Malaysia is one of the countries with the highest lightning flash density globally. While sufficiency of lightning protection system is crucial to ensure human safety against lightning strikes, the public awareness towards lightning safety is also equally important in Malaysia. Hence, this study was conducted to understand the current lightning safety awareness level of the Malaysian population.

**Methods**: An online questionnaire survey which consists of 22 scientific statements of lightning was first developed in Malay and English. The questionnaire allows the respondent to also check their own score upon completion of the questionnaire. It was then distributed to the public for data collection. The sample size comprised of both genders, all layers of society from various educational level and social background.

**Results**: Overall, the awareness on lightning safety amongst Malaysian is at moderate level with an average score of slightly above 50%. Urbanites scored marginally better than their rural counterparts. One’s education level does not dictate their awareness level of lightning safety.

**Discussion**: In conclusion, the public in Malaysia needs to be better educated on lightning safety. Similar studies should be replicated in other countries experiencing similar levels of lightning activity to better understand the public’s perception on lightning.

## Introduction

Malaysia is in the top three in the world with high lightning density experiencing an annual mean lightning ground flash density of 13.9 flashes per square kilometre yearly ^1, 2^. A recent study stated that a factor that probably contributes to the high numbers of thunderstorm and lightning events in Peninsular Malaysia is due to its geographical position being encircled by the Andaman Sea, Sulu Sea, Straits of Malacca and South China Sea ^3^. Undeniably, the other substantial factors are the massive increment of factories, deforestation and other development progress. All these activities and factors are contributing towards heating of the Earth thus increasing the severity and number of thunderstorms.

As many as 131 deaths and injuries have been reported due to lightning strikes, with 92 death injury rates per million per year. There were 22 fatalities per year from 2008–2011 reported in 4, 5. A study recently stated that lightning had killed an average of one in 10 victims in Malaysia and 235 were either killed or injured from 2008 to 2015 ^2^. These unfortunate statistics could be attributed to the weak public awareness of lightning among Malaysians. Thus, understanding lightning safety is necessary to keep them safe during the phenomena.

Two recent research were conducted to understand the public awareness level of lightning safety ^1, 6^. These studies have considered numerous sociological characteristics. However, the sample size of the previous study in 1 is not representative of the Malaysian population. Furthermore, it would be advantageous for the participants in the survey to also know their misconception towards lightning safety upon completion of the survey. Thus, this research was conducted on a larger scale to not only understand the Malaysian public’s conception of lightning safety but also attempt to educate the respondents on their misconceptions towards lightning.

## Methods

Firstly, the questionnaire was designed online in Google Form and was made bilingual, *i.e.* in Malay and English, to provide optimum understanding to respondents from different backgrounds. The questionnaire was adapted from recent surveys and interview questions in 1, 6. However, they have been further enhanced to consist of 22 questions which are grouped into two general knowledge questions, eight scientifically unaccepted statements and 12 scientifically accepted statements about lightning awareness. Respondents had to select one answer from three choices of answers namely disagree, undecided and agree. Unlike the previous studies in 1, respondents would now be able to view their scores and correct their misconceptions upon completion of the survey.

Next, the survey was randomly distributed to the Malaysian public without bias using a probability sampling approach so that everyone has an equal possibility to be selected. This approach is critical to prevent population sample size bias. A minimum of 1000 respondents is targeted as sample size based on the methodology in https://news.gallup.com/poll/101872/how-does-gallup-polling-work.aspx ^7, 8^. This targeted sample size is also in accordance with the methodology proposed by Krejcie and Morgan to determine sample size based on a confidence level of 95% and a variability of 50% for an estimated Malaysian population of 32.7 million ^9, 10^. The questionnaire was distributed randomly and was kept active until the minimum respondents is received. Each respondent was only allowed to attempt the survey once. A total of 1062 responses were received from 9 ^th^ December 2020 until 6 ^th^ January 2021. The survey was distributed to citizens aged above 18 years old from various social and educational backgrounds with their anonymity preserved. Their responses were analysed by organising the data into three parts namely age, level of education, and residency. There are three levels of age, seven levels of education, and four types of residency.

## Results

The questionnaire started with three questions to understand the level of exposure of the respondents to lightning effects. From Table 1, only 3.3% responded that they have been injured by lightning before and 9.3% have met person injured by lightning. However, 38% of the respondents reported that their home has been affected by lightning. This number seems to complement the findings in ^1^ in that the damage due to lightning is significant in Malaysia. Note that only 31.5% of the respondents consistently follow weather forecasting on television and radio; 55.5% only occasionally, and 14.9% do not follow the weather forecast at all.

**Table 1.** Respondents’ exposure to lightning effects.

Have you been injured by lightning?	
Yes	35
No	1027
Have you met a person injured by lightning?	
Yes	99
No	963
Has your home been affected by lightning?	
Yes	401
No	446
Maybe	215

The rest of the questionnaire is divided into sections A, B and C. Section A which consists of two general knowledge statements with the aim to gauge the basic understanding of lightning among the respondents. The remaining Sections B and C aim to gauge the respondents’ awareness on the nature and safety aspects of lightning. There are eight scientifically unaccepted statements in Section B and 12 scientifically accepted statements in Section C as shown in Table 2. Scientifically accepted statements means scientifically acceptable facts based on present day knowledge and understanding of lightning. In the questionnaire, the sequence of these 18 statements are randomised to ensure that the respondents could not “guess” the grouping of the statements. The participants have to select either disagree, undecided or agree for each statement.

**Table 2.** Responses received for the 22 statements.

		Number of Responses		
		Disagree	Undecided	Agree
Section A: General knowledge				
1	Lightning is four times hotter than the Sun.	176	470	416
2	Do you agree with the statement, Malaysia is known as Crown of Lightning worldwide?	149	410	503
Section B: Scientifically unaccepted statements				
1	Lightning caused by supernatural powers.	772	200	90
2	Thunder is a sign that God is angry.	630	300	132
3	Lightning victims are people with bad luck.	703	209	150
4	Assuming you are out in the open during thunderstorms with nowhere to take shelter, lie flat on the ground.	363	383	316
5	Lightning never strikes the same place twice.	415	478	169
6	If you are in a house, you are 100% safe from lightning.	433	314	315
7	If thunderstorms threaten while you are outside playing a game, it is okay to finish it before seeking shelter.	833	162	67
8	If it’s not raining or there aren’t clouds overhead, you’re safe from lightning.	478	315	269
Section C: Scientifically accepted statements				
9	Lightning is a flow of electricity.	115	182	765
10	When thunder roars, stay indoors and away from windows.	100	198	764
11	CPR can help lightning victims to survive.	467	431	164
12	During thunderstorms, you should keep at least 3m distances away from trees/fences.	100	265	697
13	Avoid having an open shower during thunderstorms.	87	185	790
14	If you hear thunder before you reach counting to 30, go indoors.	241	406	415
15	Suspend activities for at least 30 minutes after the last clap of thunder.	167	330	565
16	Do avoid open areas during the thunderstorms.	84	133	845
17	Stay away from concrete floors or walls during thunderstorms.	362	426	274
18	Kuala Lumpur is ranked 5th in the world with high lightning density.	89	623	350
19	Lightning kills 1/10 victims in Malaysia.	153	532	377
20	It is dangerous to take a swim in a river in thunderstorms.	87	182	793

In section B, the first three statements were adopted from 1. Over 50% of respondents believed a supernatural power is behind a lightning strike ^1^. However, in the present study with a much larger sample size, only 27% has similar suspicion. The responses were evenly distributed for statements 4 and 6. Majority of the respondents is aware that they should immediately cease their outdoor activities when there is thunderstorm as reflected in statement 7. In section C, statements 9–15 were adopted from 1. About 28% of the respondents are confused about the lightning’s electrical nature and this seems to concur with 1. Statement 10 came from a famous slogan from the United States and statement 14 is based on the 30–30 rule ^11^.

Overall, the majority of the respondents agreed with the scientifically accepted statements except for statement 11, 17, 18, and 19. The fact that the majority did not believe CPR can help lightning victims is worrying because it seems to suggest that the public is not prepared for any emergency arising from lightning struck victims. Statements 18 and 19’s results show that respondents are not aware of lightning issues in Malaysia.

## Discussion

In this section, the respondents’ awareness level will be analysed according to their age group, education level and residency. This awareness level is quantified by the marks that they scored. Note that the respondent will be given 1 mark for every correct response to the statements in Table 2. Hence, the maximum mark that they can score is 22.

Table 3 shows the responses which are categorized according to the respondents’ age. There is only slight difference in their understanding level when observed across the three age groups.

**Table 3.** Responses according to age.

Age group	Number of responses	Average mark
Youth (18-30 y/o)	740	11.6
Adult (31-59 y/o)	315	11.7
Senior citizen (above 60 y/o)	7	12.0

Table 4 shows the responses which are categorized according to the respondents’ education level. The findings suggest that a higher education level does not necessarily means a higher level of awareness and lightning safety knowledge.

Table 5 illustrates the responses grouped according to the residencies of the respondents. As observed here, respondents living in metropolitan areas have the highest awareness of lightning safety. However, the difference is only marginal.

**Table 4.** Responses according to education level.

Highest education level	Number of responses	Average mark
Primary School	49	11.5
PMR/PT3	45	11.9
SPM	144	12.1
Pre-University	323	11.5
Bachelor’s Degree	436	11.6
Master’s Degree	48	12.0
Doctor of Philosophy	17	10.8

**Table 5.** Responses according to residency.

Residency	Number of responses	Average mark
Village (Kampung/Luar bandar)	344	11.06
Town (Pekan)	234	11.44
City (Bandar)	349	11.91
Metropolis (Iskandar Malaysia, Kota Kinabalu, Kuala Lumpur, Kuching, Klang Valley, dan Seluruh Pulau Pinang serta Selatan Kedah serta Barat Laut Perak)	135	12.67

All in all, on the average, the respondents could only get half of the maximum score which clearly indicates the lack of awareness. Finally, Table 6 summarises the common misconceptions on lightning safety among the respondents. This could perhaps serve as a guide for relevant parties promoting lightning safety awareness in Malaysia.

**Table 6.** Summary of misconceptions.

No.	Misconception
1	Thunder is a sign that God is angry.
2	Lightning never strikes the same place twice.
3	When a person in an open area during a lightning event and nowhere to take shelter, they should lie flat on the ground.
4	If a person is in the house, they are 100% safe from lightning.
6	If there is no clouds and rains, a person is safe from lightning.
7	Lightning can pass through concrete floors and walls.
8	Lightning did not strike the same place twice.
9	During thunderstorms, one is safe if there stay near trees or fences.
10	CPR is not able to save lightning’s victim.

**Figure 1.**
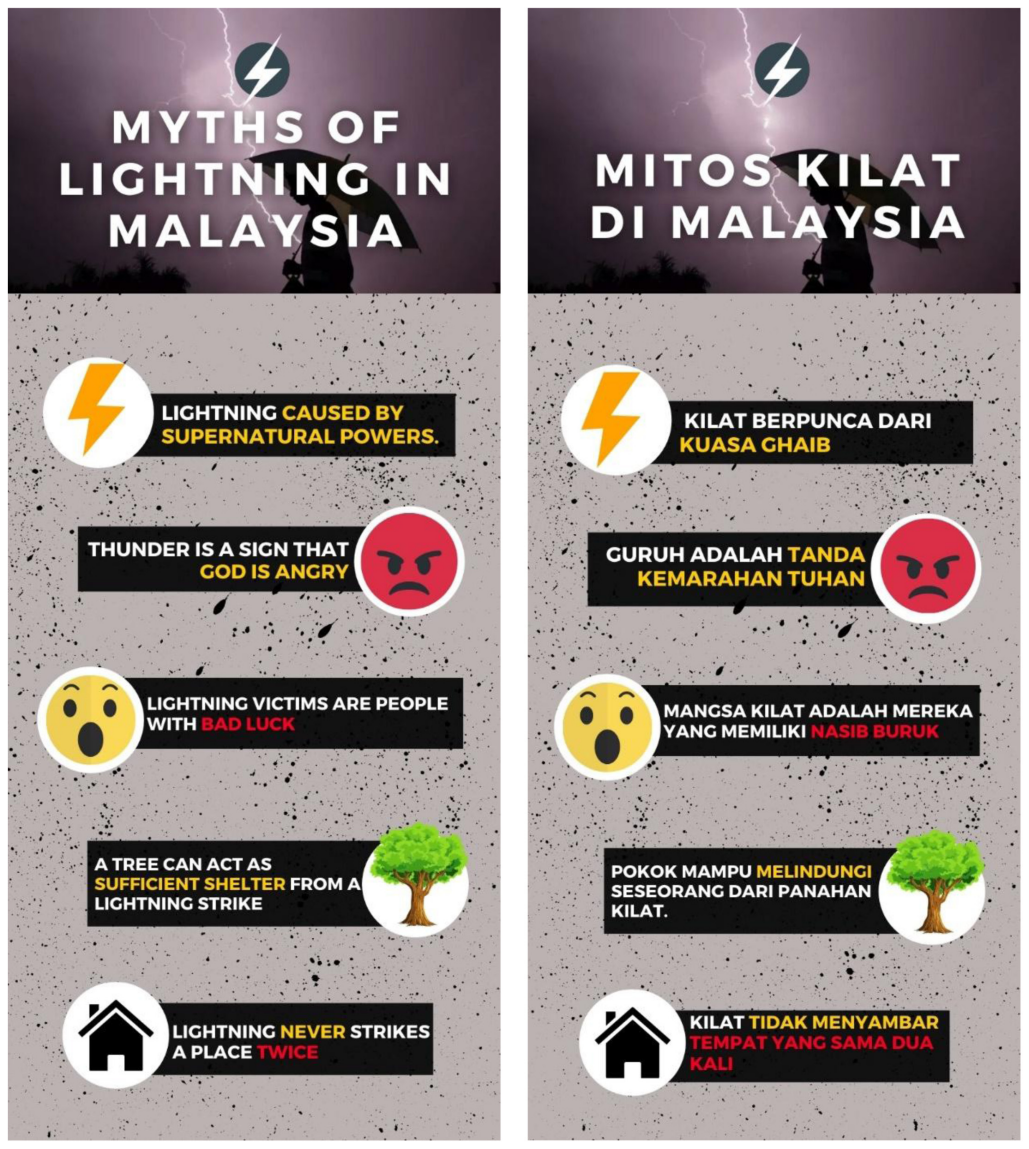
Common myths of lightning in Malaysia (in English and Malay).

**Figure 2.**
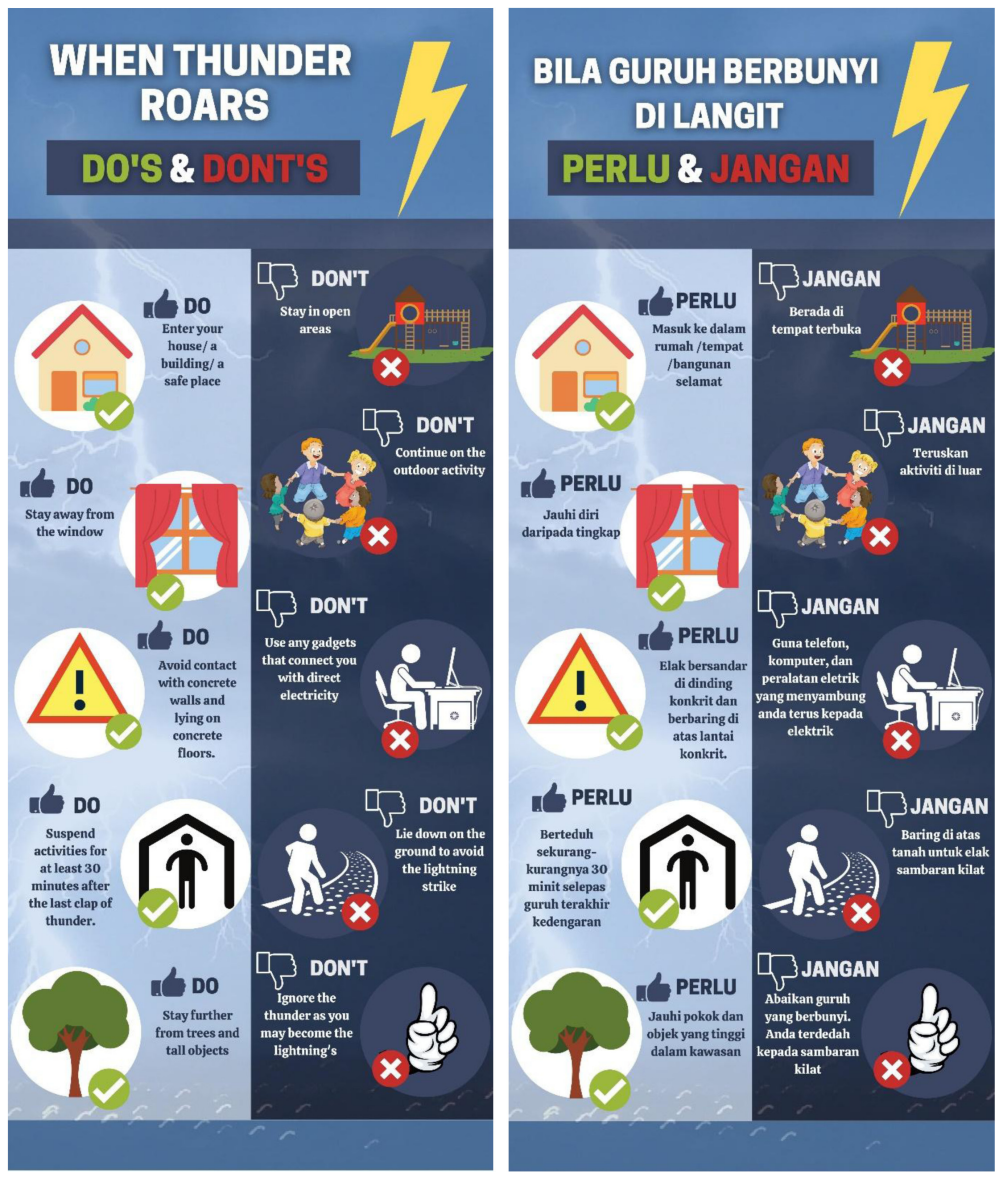
Lightning safety message based on common myths observed (in English and Malay).

Figure 1 illustrates the summary of common myths among the Malaysian public in an infographic format. On the other hand, Figure 2 presents the do’s and don’ts when there is thunderstorm which was developed based on the common myths observed in this study. Note that both infographics are available in English and Malay language.

## Conclusions

To summarize, the public awareness of lightning safety in Malaysia is moderate, proven by the number of misconceptions that existed through their responses. In the same context, their knowledge of dealing with the lightning situation is worrying. Many did not believe in the capability of CPR to save a lightning victim. From here, note that the majority will be confused about what to do if a lightning incidence happens. Furthermore, one’s level of education has little impact on their awareness of lightning safety. Moreover, urbanites in particular metropolis citizens have a better awareness of lightning safety than others.

On the average, 53% agreed with the scientifically accepted statements, and 54% disagreed with the scientifically unaccepted. The fact that the average mark of all respondents is barely half of the maximum mark means that the awareness level is still unsatisfactory. Relevant parties such as the Energy Commission and perhaps the Ministry of Education can collaborate to enhance national lightning safety education and promotion by utilising the findings in this paper. Lightning safety education campaign in Malaysia should ideally be as progressive as those in Sri Lanka, Colombia and the United States. It would also be interesting for similar studies to be replicated in other countries as well to gain a better understanding at the global level.

## Data availability

Data are available at:

Siow, Dr S.C. LIM (Multimedia University) (2021): Lightning Safety Awareness Level in Malaysia. DANS. https://doi.org/10.17026/dans-zut-4u2s.

Figures are available at:

Chun Lim, Siow; Gomes, Chandima; Nazli, Khairul (2021): Malaysian Public Awareness of Lightning Safety. figshare. Figure. https://doi.org/10.6084/m9.figshare.16768060.v1.

Data are available under the terms of the Creative Commons Zero “No rights reserved” data waiver ( CC0 1.0 Public domain dedication).

## Ethics and consent

This survey had obtained approval number of EA2152021 from Research Ethics Committee of Multimedia University.
