# Supplementary material for: Lightning safety awareness level in Malaysia
Source: F1000Res. 2021 Nov 4;10:921. Originally published 2021 Sep 15. [Version 2] doi: 10.12688/f1000research.73064.2 (PMC8596182; doi:10.12688/f1000research.73064.2)
Supplement: Peer review history

# Peer review: Reviewer response for version 2

**DOI:** 10.5256/f1000research.78307.r99036

**Published:** 2021-11-16

**Authors:** Norhidayu Binti Rameli

**Affiliations:** 1 Faculty of Engineering and Built Environment, Universiti Sains Islam Malaysia, Nilai, Negeri Sembilan, Malaysia

There are no further remarks to be made. The author answered each comment and briefly explains it.

Is the work clearly and accurately presented and does it cite the current literature?

Yes

If applicable, is the statistical analysis and its interpretation appropriate?

Partly

Are all the source data underlying the results available to ensure full reproducibility?

Partly

Is the study design appropriate and is the work technically sound?

Yes

Are the conclusions drawn adequately supported by the results?

Partly

Are sufficient details of methods and analysis provided to allow replication by others?

Yes

Reviewer Expertise:

Lightning and High Voltage Power Cable.

I confirm that I have read this submission and believe that I have an appropriate level of expertise to confirm that it is of an acceptable scientific standard.

# Peer review: Reviewer response for version 1

**DOI:** 10.5256/f1000research.76687.r94364

**Published:** 2021-10-04

**Authors:** Norhidayu Binti Rameli

**Affiliations:** 1 Faculty of Engineering and Built Environment, Universiti Sains Islam Malaysia, Nilai, Negeri Sembilan, Malaysia

This paper is about understanding the current lightning safety awareness level of the Malaysian population. The methodology surveyed 1062 respondents and included 22 questions about their level of lightning safety awareness. Additionally, the conclusion drawn from Malaysia's population is that it is of a moderate awareness level. The author and team have made an admirable effort. Nonetheless, the following points may be considered for improvement;

 1. The work's methodology should be more detailed and justified.

- It is necessary to justify the minimum number of respondents. Is this enough to represent the Malaysian population? Kindly show the evidence to support the number of respondents.
- Is there any other evidence to support the choice of the 22 questions?
- Please demonstrate how the probability sampling method is implemented.
- Any software tools that authors use to analyse the result?

2. To provide a more meaningful view, the result should be presented in an infographic such as a pie chart or histogram.

 3. The interpretation of the results discussed in this section should be based on **statistical inference** to draw conclusions about a population (Please take note that the Malaysian population is about 32 million). As a result, the conclusion should be revised appropriately.

Is the work clearly and accurately presented and does it cite the current literature?

Yes

If applicable, is the statistical analysis and its interpretation appropriate?

Partly

Are all the source data underlying the results available to ensure full reproducibility?

Partly

Is the study design appropriate and is the work technically sound?

Yes

Are the conclusions drawn adequately supported by the results?

Partly

Are sufficient details of methods and analysis provided to allow replication by others?

Yes

Reviewer Expertise:

Lightning and High Voltage Power Cable.

I confirm that I have read this submission and believe that I have an appropriate level of expertise to confirm that it is of an acceptable scientific standard, however I have significant reservations, as outlined above.

# Response

**Published:** 2021-10-08

**Authors:** Chun Lim Siow

**Affiliations:** Multimedia University, Malaysia

**Comment:**

 This paper is about understanding the current lightning safety awareness level of the Malaysian population. The methodology surveyed 1062 respondents and included 22 questions about their level of lightning safety awareness. Additionally, the conclusion drawn from Malaysia's population is that it is of a moderate awareness level. The author and team have made an admirable effort. Nonetheless, the following points may be considered for improvement;

 1. The work's methodology should be more detailed and justified.

- It is necessary to justify the minimum number of respondents. Is this enough to represent the Malaysian population? Kindly show the evidence to support the number of respondents.

**Response:**

 Thank you for the comment. We followed the approach in the Gallup poll where the typical sample size is 1000 national adults to represent the opinion of the population of a given country with a margin of error of ±4%. We have also verified the sample size using the methodology proposed by Krejcie and Morgan (1970) to determine sample size based on a confidence level of 95% and a variability of 50%. 

**Comment:**

- Is there any other evidence to support the choice of the 22 questions?

**Response:**

 Thank you for the comment. Based on available literature, the most comprehensive questionnaire is those available in Syakura *et al.* (2019), which we have adapted from. However, we have slightly enhanced the questionnaire by adding 5 more questions. In addition, the respondents are also able to know their score upon completion of the questionnaire. This is a side objective of this work to also educate the respondents on lightning safety.

**Comment:**

- Please demonstrate how the probability sampling method is implemented.

**Response:**

 Thank you for the comment. The questionnaire was randomly distributed to citizens aged above 18 years old. Respondents are only allowed to respond once.

**Comment:**

- Any software tools that authors use to analyse the result?

**Response:**

 Thank you for the comment. Microsoft Excel is used to analyse the result.

**Comment:**

 2. To provide a more meaningful view, the result should be presented in an infographic such as a pie chart or histogram.

**Response:**

 Thank you for the comment. We totally agree with your suggestion that a pie chart or histogram enhances the clarity. However, presenting the data in a table also has its own merit as it allows readers to conveniently extract the raw data. To provide a meaningful view and further enhance the visibility of the findings, we have included 2 infographics (Figure 1 and Figure 2) which are also accessible here.

**​​​​​​​Comment:**

 3. The interpretation of the results discussed in this section should be based on **statistical inference** to draw conclusions about a population (Please take note that the Malaysian population is about 32 million). As a result, the conclusion should be revised appropriately.

**Response:**

 Thank you for the comment. We have explained how we arrived at the sample size of 1000 in our earlier response to your earlier comment above. While we did not claim that the findings reflect the opinion of the Malaysian population, the sample size used in this study is actually statistically valid assuming 95% confidence interval for a 32 million population.

# Peer review: Reviewer response for version 1

**DOI:** 10.5256/f1000research.76687.r94361

**Published:** 2021-10-04

**Authors:** Helio Eiji Sueta

**Affiliations:** 1 Planning, Analysis and Energy Development Scientific Division of the Energy and Environment Institute (IEE-USP), University of São Paulo, São Paulo, Brazil

In principle, it is not a “purely “scientific article with dozens of formulas and/or complex mathematical simulations, but nevertheless it is a very important article. I comment on this because the vast majority of technical papers published in scientific journals bring theoretical or experimental developments with great mathematical formulations and models. In this paper we find a very well done analysis of the responses to a very well-designed survey for a given population. An advantage of not being “purely” scientific is that it can be read by any type of person, regardless of their area of expertise, as in addition to having a very accessible language, the subject is of general interest to all areas. I don't see any disadvantages in this fact.

 I fully agree with the author that in many countries, especially developing ones, the general knowledge of the population about the dangers of lightning is very limited and, many times, surrounded by myths. The work presents the results of a survey to the population about general aspects of lightning, mixing some scientifically accepted statements with others not accepted. The results were quite interesting, showing that in Malaysia, where the research was applied, the general population still has many failures in awareness of the dangers of lightning. It also showed that this awareness is a little better in urban centers than in rural ones, but that better school education is not significant for a better awareness of this issue.

 The work fulfills what was proposed and can be replicated in other countries. As a suggestion, it would be interesting to compare the results of the same survey in several countries, including well-developed countries, in order to better understand the general awareness of the world population about the dangers involved in lightning.

Is the work clearly and accurately presented and does it cite the current literature?

Yes

If applicable, is the statistical analysis and its interpretation appropriate?

Yes

Are all the source data underlying the results available to ensure full reproducibility?

Yes

Is the study design appropriate and is the work technically sound?

Yes

Are the conclusions drawn adequately supported by the results?

Yes

Are sufficient details of methods and analysis provided to allow replication by others?

Yes

Reviewer Expertise:

I am currently the deputy head of the Scientific Division of Energy Planning, Analysis and Development at the Institute of Energy and Environment at the University of São Paulo. My main research area is the protection of structures and people against lightning strikes. I am currently the secretary of the Brazilian Committee that reviews the lightning protection standard.

I confirm that I have read this submission and believe that I have an appropriate level of expertise to confirm that it is of an acceptable scientific standard.

# Response

**Published:** 2021-10-08

**Authors:** Chun Lim Siow

**Affiliations:** Multimedia University, Malaysia

Thank you for the comments. Indeed, we need more similar kind of work to be done especially in developing or less-developed countries to gain a holistic understanding of how the public perceives lightning. With these findings, relevant stakeholders (policymakers, academia etc) can then strategise a more targeted lightning awareness promotion approach to minimise unacceptable loss of human lives due to a natural phenomenon which we have already heavily researched on for more than a century.
